# Case of Death by Poisoning with the Leaves of Aconite or Monkshood, Passed off in Jest for Parsley

**Published:** 1844-09-21

**Authors:** Al. Ramsay

**Affiliations:** Surgeon


*Case of Death by Poisoning with the Leaves of Aconite or Monkshood, passed off in jest for Parsley.* By Al. Ramsay, Surgeon.

At about 11 A. M., a boy named Hanton, aged 14, on passing a garden in the parish of Murroes, looked over the wall, and asked a young man in the garden, "if he would give him some parsley;" to which it was answered, that he would get some new parsley, as being much better than the old kind. The young man accordingly gave Hanton a handful of green leaves, of which he ate some. In about two hours after, he complained of a burning sensation in the mouth, throat, and stomach, and was very sick. For this his mother made him take a glass of whiskey. Some time after this he took a fit and fell on the ground—at 6 o'clock P. M., on his mother returning from her work, she found him lying across the bed, with his hands in his pockets, dead and stiffening. At the post mortem the blood-vessels within the head were found enormously distended with dark-coloured fluid blood; upwards of a pound of which escaped from the skull and spinal canal. The stomach was empty, with a deep, inflammatory blush over its whole internal surface, and here and there patches of a darker colour—further dissection was not allowed.

Death appears in this case to have been caused by the apopletic state of the brain, probably ending in asphyxia.—*North. Jour. of Med.*

